# An investigation on main effects and interactions of relative age effects and playing position in female elite football in Germany

**DOI:** 10.3389/fspor.2024.1459784

**Published:** 2024-10-03

**Authors:** Kristy L. Smith, Till Koopmann, Patricia L. Weir, Jörg Schorer

**Affiliations:** ^1^ Department of Kinesiology, University of Windsor, Windsor, ON, Canada; ^2^ Institute of Sport Science, Carl von Ossietzky Universität Oldenburg, Oldenburg, Germany

**Keywords:** athlete development, constant year, within year, between year, birth advantage, competition structure, age cohort

## Abstract

Athlete age-grouping and age-banding has been shown to impact sport participation and athlete development. The current study examined the impact of within-year (WYEs) and between-year effects (BYEs), and their interactions with playing position, on female participation in elite German football. The sample of 1,378 German first-league players revealed expected participation inequities within-year with relatively older players being over-represented and younger players being under-represented. From a between-year perspective there were no participation differences. The interaction of WYEs and BYEs revealed an over-representation of odd-year players in Q1, and Q2 in even years. With respect to the interaction between year effects and playing position, for WYEs the expected effect was present for goalkeepers and defenders, while there was no significant interaction for BYEs. Overall, the results offer insight regarding the impact of system structure on participation, and highlight unique characteristics associated with playing positions.

## 1 Introduction

Research examining sport-related relative age effects (RAEs) is concerned with training, competition, and selection (dis)advantages experienced by members of the same age-grouped cohort. When examined within the context of sport, RAEs are assumed to be present when patterns of over-/under-representation are observed with respect to the participants’ birthdates and their respective relationship to the organizational cut-off date used for age groupings (1). To illustrate the effect within a one-year cohort, an individual born in January (i.e., relatively oldest) may be more likely to participate and advance in certain sport settings than an individual born in December (i.e., relatively youngest) due to inherent chronological age differences (2). RAEs are most commonly investigated as *within-year effects (WYEs)* when examined within the same birth year but can also include (dis)advantages experienced by athletes grouped into two-year age bands (i.e., *between-year effects; BYEs*) where disadvantages will be more pronounced. For instance, at age 12 children can vary by 22.5 cm (girls) or 24.4 cm (boys) with respect to attained height, and by 28.2 kg (girls) or 25.7 kg (boys) for weight based on longitudinal data (3); the size range would be even greater within a two-year age band. Here it is important to note that RAEs can become exacerbated in combination with a second, closely connected and interacting but different factor/bias influencing player development: variations in biological maturity^1^ (4, 5). A variety of RAEs have been documented across sport and cultural contexts over the years (for reviews, see 6, 7). Generally, patterns of over-representation of relatively older athletes are found at the developmental level in both team-based (e.g., soccer, ice hockey) and individual (e.g., tennis, swimming) sport contexts, particularly when physical demands are an inherent element of the activity (6, 7).

### 1.1 RAEs in sport

The magnitude of RAEs can vary depending on age level, competitive tier, and sex/gender (8). For instance, existing meta-analyses have found that RAEs in male athlete samples peak during the adolescent years (6) when physical differences between peers are most extreme due to developmental processes (9, 10). In (female) athlete samples, the magnitude of RAEs peak earlier before dissipating (7), reflecting differences in biological growth (11) and earlier completion of maturation processes (i.e., typically 12–14 and 13–15 years for females and males, respectively 8). Furthermore, RAEs are believed to be more common when physical development/size provides a competitive advantage in a given sport (10), and when athlete selection processes (particularly at young ages) or competition for a small number of playing positions are inherent in the organizational structure of the sport (12). These trends align with the frequently cited ‘maturation-selection’ hypothesis [see (13, 14) for further discussion]; however, multiple factors could potentially contribute to the magnitude and nature of these effects during the course of development, e.g., socio-cultural factors [for further discussion, see (10, 15, 16)].

Typically, RAEs are seen within one year across a single cohort (WYEs). In sport contexts, athletes often participate in an age-grouping category and a competition age-band (e.g., under-16) simultaneously. When athletes enter year one of a multi-year (mostly two-year) age-band they are members of an age-group. When this group progresses to year two, a new annual age-group enters year one of the age-band. In this situation, each year of the age-band represents a *constituent year* where a player can be in the younger year group in year one of an age-band and in the older year group in year two of the age-band, with relative age being a dynamic characteristic (1, 17–19). That is, constituent year effects represent one type of BYEs and it is the interaction of WYEs and BYEs that has provided an opportunity to look at athlete development in new ways. The other type of BYEs are *constant year effects* where multi-year age-groups are fixed and constant throughout development, and all players move together from one age-band to the next. In this system, the oldest players remain the oldest across the different age-bands and conversely the youngest players are always the youngest, and may consistently be at a disadvantage. In some cases, like in the following study, constant year effects and constituent year effects can interactively affect the same sample simultaneously when two development systems (e.g., the club and German national talent development system) are working in parallel.

### 1.2 Interaction of BYEs and WYEs

Previous work examining both WYEs and BYEs simultaneously has been limited, with some variation in the observed trends depending on the context (e.g., team vs. individual sport) and age group (e.g., junior vs. senior/professional). For instance, Steingröver and colleagues (20) examined WYEs and BYEs at the highest level of (male) German U16 and U19 basketball, observing a statistically significant interaction in the U16 division, and statistically significant main effects for both effects in the U19 age group. Likewise, Steingröver et al.'s (21) study revealed an interaction of WYEs and BYEs across a sample of (male) soccer players competing in U17 world cup tournaments, suggesting that younger players are less likely to enter the national athlete development system. However, there did not appear to be a carry-over effect in a subsequent sample of world cup athletes, although some WYEs were replicated. Moreira and colleagues (22) reported the presence of both WYEs and BYEs with respect to junior tennis players’ rankings by the International Tennis Federation, with 19% of the variance explained by the presence of both effects. For national and international table tennis, no interaction was found between WYEs and BYEs, but various subsample analyses suggested main effects (17). In summary, the first studies investigating the interaction of WYEs and BYEs all show main effects for both WYEs and BYEs while an interaction is only rarely found.

### 1.3 Interaction of playing position and with WYEs or BYEs

Previous research has also examined the influence of playing positions as a moderator of RAEs (8, 23). Several studies have documented variation in magnitudes of *WYEs* depending on playing position within this context. For instance, Baker and colleagues (24) documented positional differences with respect to WYEs in a male/female, youth and adult sample competing at the national level for USA soccer. Playing position has also been identified as a moderator in other sport contexts, such as ice hockey (e.g., 25) and handball (e.g., 26). While variation is observed in the soccer/football-related findings published to date, *WYEs* generally tend to be greater in magnitude among subsamples designated as defense or goalkeepers (24, 27–29), and also for the midfield position (24, 27, 30) when birth distributions are compared across position categories within a sample.

For BYEs, there are to the best of our knowledge no studies that analyzed the interaction of BYEs and playing position. Therefore, with respect to soccer/football, moderation of *BYEs* with respect to playing position remains unexamined.

### 1.4 Aim of the study

The complexity of WYEs and BYEs operating simultaneously (both uniquely and as interacting variables) in sport development systems remains largely unexamined, particularly in female contexts. Thus, the aim of this study was to investigate (1) both WYEs and BYEs in combination with the playing position, and (2) their possible interaction among elite female athletes competing in German league soccer.

For the WYEs, a clear over-representation of the first quartile in comparison to other birth quartiles was hypothesized. For the BYEs, the hypothesis is less straight forward, because the German female football system comprises of two different development systems. On the one hand, the national development system is a constant year system with fixed two-year age bands, with the relatively older year group constantly being the older ones. On the other hand, the club development system acts on a constituent year approach in which the seniority changes every year. Here, player groups are the older one for one year and the next year they are the younger one. All players will participate in the club system, while only some will make it into the national development teams. Therefore, a clear hypothesis in this regard cannot be stated and this effect was explored openly. For playing position, current tactical systems in soccer/football require a different number of players per position and therefore, clear differences were expected (i.e., less goalkeepers than strikers, than defenders and midfielders).

For the interaction of BYEs and WYEs, only very limited empirical studies can be found in the current literature (20, 21). Thus, this investigation was exploratory without clear hypotheses as previous research investigated only younger age divisions. For the interaction of playing position and WYEs, statistically significant effects were hypothesized as previous research has shown variable WYEs, or more general RAEs, magnitudes for different playing positions although the interaction was not specifically tested in those studies. No specific hypotheses were generated for playing position given the novelty of this portion of the analysis with respect to BYEs.

## 2 Methods

### 2.1 Data acquisition and processing

For this study, birthdates and playing positions of 1,387 players were obtained from official websites covering the first German football league (https://www.kicker.de/frauen-bundesliga/vereine/). The players were born between 1977 and 2001. Their mean age was 24.32 years. For the German Football Association, January 1st was defined as the cut-off date. For WYEs, birth dates were recoded into four quartiles (Q1: January–March, Q2: April–June, Q3: July–September, Q4: October–December). For BYEs, birth years were divided into odd and even years. However, as mentioned above, these odd and even years cannot be attributed as older or younger because of the changes in the club system. For playing position, players were divided into groups of goalkeepers, defenders, midfielders, and strikers. In this study, we included only German-born players, because the developmental systems all over the world might be different from the German structure.

### 2.2 Statistical analyses

IBM SPSS Statistics 29.0 and G*Power 3.1 were used for statistical analyses (31). Asymptotic chi²-analyses were conducted to (1) consider differences among birth quartiles (WYEs), (2) test for differences among the number of participants in odd or even years, respectively (BYEs), and (3) test for differences in number of players per position. In addition, asymptotic chi²-analyses were conducted to test for two-way interactions between the three factors. For all analyses, an equal distribution of births across all quartiles and years was assumed and the alpha-level was set at .05. For each effect size, the 90% confidence interval was calculated based on the noncentral chi²-files provided online by Michael Smithson (http://www.michaelsmithson.online/stats/CIstuff/CI.html).^2^

## 3 Results

The results section follows the two aims of the study. First, single factor effects were checked for WYEs, BYEs, and playing position effects. Second, two-way interactions between the three factors were reported.

**Figure 1:**
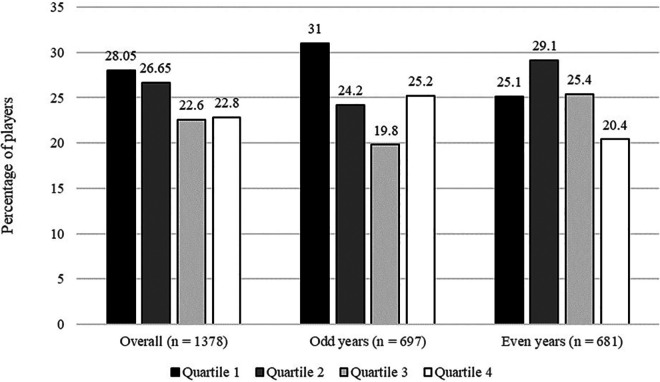
Interaction of BYEs and WYEs.

For WYEs, a statistically significant effect of small size was revealed, *χ*²(3, *n* = 1,378) = 12.54, *p* < .01, *w* = .10, 90%CI[.03; .14]. In the first quartile, 28% of players were born and therefore the first quartile was over-represented in comparison to the other quartiles (Q2 = 26%; Q3 = 23%, and Q4 = 23%; cf. Figure 1). For BYEs, no statistically significant effect was found, *χ*²(1, *n* = 1,378) = 0.19, *p* = .67, *w* = .01, 90%CI[.00; .06], with 51% of players being born in the odd years and 49% of players in even years. For the factor of playing position, a statistically significant effect of medium size was found, *χ*²(3, *n* = 1,378) = 181.07, *p* < .01, *w* = .36, 90%CI[.31; .41]. As expected, there were fewer goalkeepers (13%) in the sample compared to strikers (20%), defenders (31%) and midfielders (36%).

The first two-way interaction analyzed was for WYEs and BYEs. As can be seen in Figure 1, for odd years the expected over-representation for the first quartile was found. For even years, Q2 had the greatest representation. A two-way chi²-analyses revealed a statistically significant interaction, *χ*²(3, *n* = 1,378) = 15.57, *p* < .01, *w* = .11, 90%CI[.04; .15]. This interaction is small to non-existent as shown by the effect size confidence interval.

**Figure 2:**
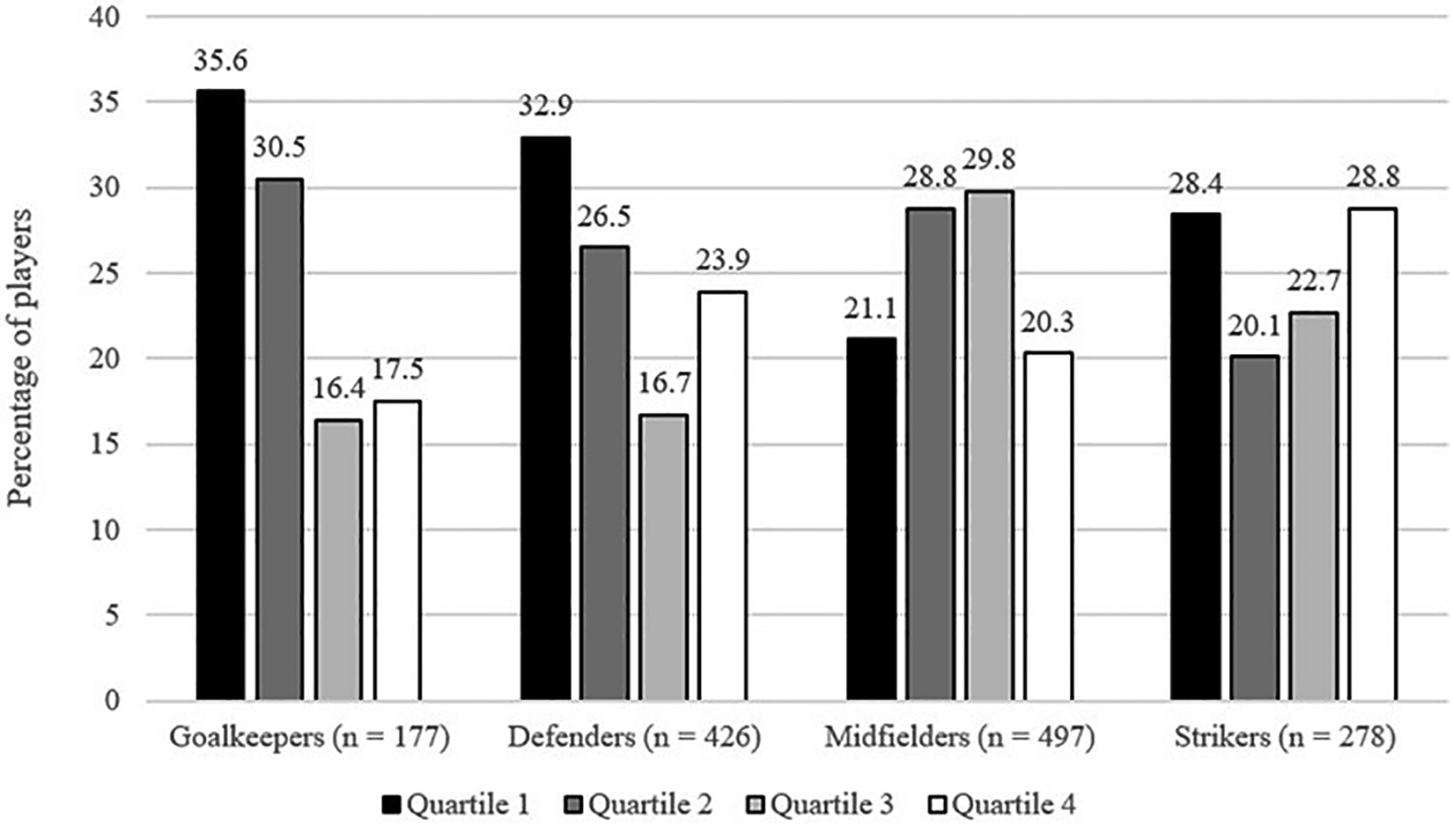
Interaction of WYEs and playing position.

For the interaction between playing positions and WYEs, a varying pattern of results were found. For goalkeepers, the expected WYE was found (cf. Figure 2). For defenders, the pattern for Q1, Q2 and Q3 was the same, but Q4 showed a higher number of players than expected. For midfielders, an over-representation for Q2 and Q3 and under-representation for Q1 and Q4 was observed. The opposite trend is demonstrated for strikers. Overall, the interaction was statistically significant with a small effect size, *χ*²(9, *n* = 1,378) = 50.99, *p* < .01, *w* = .19, 90%CI[.12; .23].

**Figure 3:**
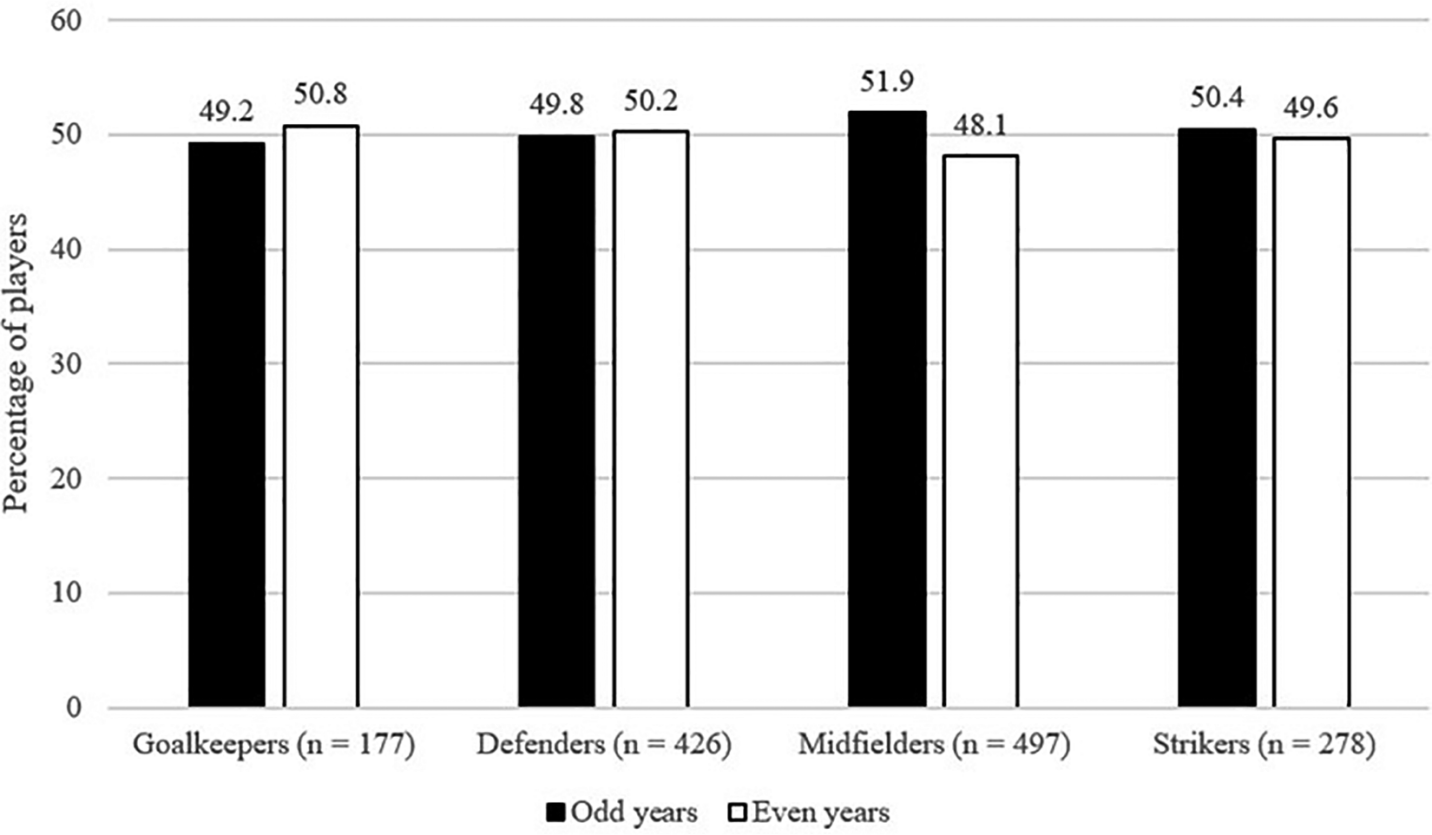
Interaction of BYEs and playing position.

For the interaction between playing positions and BYEs, similar patterns of results were found as with WYEs (cf. Figure 3). As described for the playing position effect, the overall number of players increased from goalkeeper to strikers, defenders, and to midfielders, however, this trend was similar for both odd and even years as shown by the effect was not statistically significant effect and the effect size was below small, *χ*²(3, *n* = 1,378) = 0.61, *p* = .89, *w* = .02, 90%CI[.00; .05].

## 4 Discussion

The purpose of this study was to examine WYEs and BYEs, their possible interaction, and playing position effects in elite female athletes competing in German league soccer.

### 4.1 Single factor effects

Findings were consistent with hypotheses for WYEs, with a statistically significant over-representation of relatively older athletes in German first-league football. However, effect sizes were small for these WYEs, because while WYEs overall mimic and support those published previously and highlighted in the meta-analysis by Smith et al. (7), the pattern of over-representation was different for odd and even years with an over-representation in Q1 for odd years and Q2 for even years.

Analyses of BYEs were exploratory in nature and suggested no significant main effects among German-born female athletes. This means that being born in the first or second year of a two-year age band was not associated with any relative advantage or disadvantage with respect to German first league participation. The lack of BYEs may be explained by the overwhelming role of the clubs’ development system (incorporating a dynamic, constituent system wherein athletes alternate being oldest and youngest from year to year) in contrast to national activities (where the year to year advantage remains fixed). It might also reflect, that not all women reaching the first league in Germany were actually part of the national talent development system. Therefore, its influence might be smaller. In essence, the BYEs may potentially be masked by players at similar stages of maturation or physical development despite the constant year system where those born in odd years are always older than those born in even years. However, additional work is needed to further explore findings related to BYEs.

Single factor analyses for playing position revealed clear differences in the number of players for each position category, as expected. This aligns with current tactical systems in football/soccer and reported findings in previous work (29, 30).

### 4.2 Interaction effects

Visual inspection of Figure 1 suggests subtle (descriptive) differences in the pattern of birth quartile representation (WYEs), when comparing odd and even years (BYEs), with the greatest percentage of players born in Q1 (odd) vs. Q2 (even). Previous analyses of female samples have shown similar trends (7). While an over-representation of Q1 is theoretically expected according to the classic patterning of RAEs, it is not uncommon for Q2 over-representation to be reported, suggesting RAEs are not as clearly defined in female samples. However, the practical significance of an interaction between WYEs and BYEs in this study is likely minimal, as indicated by the effect size.

With respect to playing position, only the interaction with WYEs was significant, supporting previous research. While all playing positions showed variation of representation across birth quartiles, only goalkeepers and defenders displayed a traditional over-representation in Q1 and under-representation in Q4. This is similar to the findings of Romann and Fuchslocher (27) who reported stronger birth quartile effects for goalkeepers and defenders in female Swiss soccer. Other studies have shown more consistent birth quartile effects across playing positions in soccer, handball and volleyball, but differences in structures of leagues and developmental systems and tactical requirements are likely responsible for study specific differences (32, 33). These tactical requirements may also partly explain why the (traditional) profile of a smaller, agile and smart midfielder and striker is not as prone to be influenced by RAE-related characteristics such as height and strength.

As can be observed in Figure 3, there was no interaction found between playing position and BYEs. While the overall number of players varied between position categories, there was no associated advantage with being born in an odd vs. even year within any position category. This too might be explained by the influence of the club development system, in which athletes experience varying relative age positions from year to year (i.e., constituent year effects). Examinations of playing position and BYEs in other sport contexts are not available for comparison.

### 4.3 General discussion

As stated in the introduction, recent research has emphasized the important differentiation between RAEs and biological maturity differences (4, 5, 34). While RAEs appear to be present from childhood on, maturity differences start occurring mainly with puberty onset and increase afterwards (5). That is, the two distinct factors certainly have interacting effects on player development before adulthood. In this regard, recent research in male soccer/football has shown that coaches have strong biases towards relatively older and maturer players when making selection and release decisions, potentially with greater impacts of maturity differences compared to RAEs (4, 35). As another example in a different sport, Papadopoulou et al. (36) examined anthropometric and physiological differences across birth quartiles in a population of female volleyball players. While they revealed strong birth quartile effects, they found no differences in anthropometric/physiological measures, suggesting and supporting the idea that not only age but also other maturation-related factors are responsible for these effects. While transferring findings of research on male players and/or other sport systems to female contexts must be done with caution, this certainly opens up promising avenues for future research.

While statistically significant effects were revealed for WYEs in the single factor and interaction analyses, the effect sizes were small to non-existent. Thus, the current data may support that those who are relatively younger and “survive” in the system are equally represented at the national level. The underdog hypothesis (37) proposes that relatively younger athletes may have the greatest potential for success in the long run. In order to be retained in the development system, relatively younger players “must either possess and/or develop superior technical, tactical and psychological skills” [p. 148 (38)]. These skills become more salient as the athlete moves through the developmental years towards adulthood. Yet, many relatively younger athletes may be lost due to the disadvantages that they experience.

### 4.4 Strengths & limitations

This study provided a novel analysis of BYEs and playing position in a female sample from the first German league, in addition to the more traditionally examined WYEs. The limitations of this study include those inherent in all cross-sectional, quantitative studies of RAEs. The patterns observed cannot be fully explained given the multitude of factors contributing throughout the athletes’ development over many years. One associated limitation in the present study is a lack of information regarding biological maturity in this sample. While RAEs and their assessment alone certainly have great value for both researchers and practitioners, maturational status has been identified as a significant contributor to selection advantages during the adolescent years (5, 34). Thus, including measures of biological maturity (e.g., Age at Peak Height Velocity) can provide additional information and facilitate the implementation of effective solutions. It is also difficult to fully disentangle the contributions of the club system (i.e., constituent year effects) and national system (i.e., constant year effects) in German football.

### 4.5 Future directions

Future studies should consider the various types of RAEs (WYEs and BYEs) interacting with other moderators (e.g., playing position, sex/gender) during the course of athlete development. Looking at the statistical significance of each analysis and effect sizes, this study shows how important it is for researchers to calculate, interpret and report effect sizes and confidence intervals. This allows for the assessment of practical relevance of findings. With RAEs and maturity differences having varying impacts at different phases of development, practical strategies to address the different factors should be considered. For example, birthday banding for RAEs (39) or bio-banding for maturity differences (40) should be implemented at the right time during development to mitigate selection biases.

## 5 Conclusions

The findings of the present study suggest the presence of WYEs in female German-born first league players, with minimal evidence of BYEs or an interaction between the two types of RAEs. Playing position was found to be an important moderator with respect to WYEs, highlighting unique characteristics for each position. No such trends were identified for playing position with respect to BYEs. Continued work is needed to fully unravel the contribution of RAEs (and maturation) on athlete selection.

## Data Availability

Publicly available datasets were analyzed in this study. This data can be found here: https://www.kicker.de/frauen-bundesliga/vereine/.

## Footnotes

^1^Biological maturity refers to the progression of bodily systems toward the adult state. Children of the same chronological age can vary significantly with respect to the stage (pre-pubertal, pubertal, or post-pubertal), timing, and rate/tempo of maturation (4, 5, 9). While RAEs are observed in childhood and continue throughout the developmental years, maturational differences are associated with selection bias during the adolescent period (5).

^2^The SPSS-script provided by Michael Smithson calculates the non-centrality parameter for the non-central distributions under which a particular chi²-value cuts off an area of α% (here: 5%) to the right (λ_low_) and left (λ_low_). Based on these calculations, the lower (*w*_low_) and upper (*w*_upp_) bound of the confidence interval on Cohen’s *w* are obtained via the formula *w*_low_=sqrt[λ_low_/N] and *w*_upp_=sqrt[λ_upp_/N], respectively.
